# The Sle1 Cell Wall Amidase Is Essential for β-Lactam Resistance in Community-Acquired Methicillin-Resistant *Staphylococcus aureus* USA300

**DOI:** 10.1128/AAC.01931-19

**Published:** 2019-12-20

**Authors:** Ida Thalsø-Madsen, Fernando Ruiz Torrubia, Lijuan Xu, Andreas Petersen, Camilla Jensen, Dorte Frees

**Affiliations:** a Department of Veterinary and Animal Sciences, University of Copenhagen, Copenhagen, Denmark; b Statens Serum Institut, Copenhagen, Denmark

**Keywords:** antibiotic resistance, ClpXP protease, MRSA, Sle1, autolysins, beta-lactams, cell division, daughter cell separation

## Abstract

Most clinically relevant methicillin-resistant Staphylococcus aureus (MRSA) strains have become resistant to β-lactams antibiotics through horizontal acquisition of the *mecA* gene encoding PBP2a, a peptidoglycan transpeptidase with low affinity for β-lactams. The level of resistance conferred by *mecA* is, however, strain dependent, and the mechanisms underlying this phenomenon remain poorly understood. We show here that β-lactam resistance correlates to expression of the Sle1 cell wall amidase in the fast-spreading and highly virulent community-acquired MRSA USA300 clone. Sle1 is a substrate of the ClpXP protease, and while the high Sle1 levels in cells lacking ClpXP activity confer β-lactam hyper-resistance, USA300 cells lacking Sle1 are as susceptible to β-lactams as cells lacking *mecA*. This finding prompted us to assess the cellular roles of Sle1 in more detail, and we demonstrate that high Sle1 levels accelerate the onset of daughter cells splitting and decrease cell size. Vice versa, oxacillin decreases the Sle1 level and imposes a cell separation defect that is antagonized by high Sle1 levels, suggesting that high Sle1 levels increase tolerance to oxacillin by promoting cell separation. In contrast, increased oxacillin sensitivity of *sle1* cells appears linked to a synthetic lethal effect on septum synthesis. In conclusion, this study demonstrates that Sle1 is a key factor in resistance to β-lactam antibiotics in the JE2 USA300 model strain and that PBP2a is required for the expression of Sle1 in JE2 cells exposed to oxacillin.

## Abstract (precis)

Most clinically relevant methicillin-resistant Staphylococcus aureus (MRSA) strains have become resistant to β-lactams antibiotics through horizontal acquisition of the *mecA* gene encoding PBP2a, a peptidoglycan transpeptidase with low affinity for β-lactams. The level of resistance conferred by *mecA* is, however, strain dependent, and the mechanisms underlying this phenomenon remain poorly understood.

## INTRODUCTION

The commensal bacterium Staphylococcus aureus, which colonizes the nasal cavities of about one-third of the human population, is a leading cause of bacterial infections, with disease manifestations ranging from superficial skin infections to life-threatening invasive diseases (1). Historically, β-lactam antibiotics have been the agents of choice for the treatment of staphylococcal infections. However, effective treatment of these infections is hampered by the rapid spread of methicillin-resistant S. aureus (MRSA) that are resistant to virtually all β-lactam antibiotics (1, 2). The emergence of community-acquired MRSA (CA-MRSA) has dramatically increased the global burden of S. aureus infections, and the CA-MRSA clone USA300 is currently the most common cause of purulent skin infections in emergency departments in the United States (2, 3). MRSA strains have acquired resistance to β-lactam antibiotics through horizontal acquisition of the *mecA* gene encoding PBP2a, an alternative transpeptidase with low affinity for most β-lactams. Hence, PBP2a is capable of performing the critical cross-linking of peptidoglycan strands when the native penicillin-binding proteins (PBPs) are inhibited by the irreversible binding of β-lactams to the active site (1). Clinically, MRSA isolates exhibit highly variable levels of resistance and specifically USA300 strains exhibit a relatively low level of resistance compared to other MRSA strains (4, 5). The molecular mechanisms underlying the strain-dependent resistance to β-lactams remain poorly understood, but the lack of correlation between resistance level and the level of PBP2a expression suggests that factors other than PBP2a are involved (6–9). One such factor is PBP4, which is required for β-lactam resistance in the CA-MRSA strains MW2 and USA300 but not in the highly resistant hospital-acquired MRSA (HA-MRSA) strain COL (4, 10). Similarly, the ClpXP protease contributes to the level of β-lactam resistance in the CA-MRSA strains USA300 but not in the HA-MRSA strain COL (5). The highly conserved cytoplasmic ClpXP protease is composed of separately encoded proteolytic subunits (ClpP) and ATPase units (ClpX), where ClpX serves to specifically recognize, unfold, and translocate substrates into the ClpP proteolytic chamber for degradation (11). Interestingly, inactivation of each of the components of the ClpXP protease substantially increased the β-lactam resistance level of the CA-MRSA USA300 model strain JE2 without changing the level of PBP2a or the muropeptide profiles of the cell wall, and the mechanism by which ClpXP proteolytic activity modulates β-lactam resistance remained unexplained (5). In S. aureus, only a few ClpXP substrates, such as the essential transcriptional regulator, Spx, and the cell wall amidase, Sle1, have been identified (12–14). Here, we report that the highly increased β-lactam resistance displayed by the USA300 cells lacking ClpXP activity is completely lost upon inactivation of *sle1*, suggesting that high Sle1 levels are causing the increased β-lactam resistance of *clpX* or *clpP* mutants. Conversely, inactivation of *sle1* rendered USA300 wild-type (WT) cells hypersusceptible to β-lactam antibiotics. These results are surprising, since the activity of cell wall hydrolases is typically associated with cell lysis following β-lactam treatment and not with promoting survival (15–18). The finding that Sle1 modulates the resistance level of USA300 JE2 prompted us to assess the role of the Sle1 cell wall amidase in S. aureus cell division in more detail. Super-resolution microscopy revealed that high Sle1 levels accelerate the onset of daughter cell separation, starting from the peripheral wall, resulting in cells of reduced size. Vice versa, oxacillin imposes a cell separation defect that is rescued by high Sle1 activity, suggesting that high Sle1 activity enhances tolerance to oxacillin by promoting daughter cell splitting. We further show that the level of Sle1 is reduced 2-fold when JE2 cells are grown in the presence of oxacillin and 10-fold if *mecA* is inactivated. Taken together, these findings indicate that expression of Sle1 is coupled to the transpeptidase activity of PBPs and that PBP2a becomes essential for Sle1 expression when the transpeptidase (TP) site of native PBPs is blocked by oxacillin. Finally, we show that the increased oxacillin sensitivity of *sle1* cells seems to be linked to a synergistic lethal effect on septum synthesis.

## RESULTS

### Disruption of the ClpP recognition tripeptide in ClpX confers β-lactam hyper-resistance in USA300.

We previously showed that deletion of either the *clpX* or the *clpP* gene resulted in a substantial increase in β-lactam resistance of the clinically important CA-MRSA clone USA300, suggesting that β-lactam resistance can be modulated via pathways depending on the activity of the ClpXP protease (5). In S. aureus, ClpP can associate with an alternative substrate recognition factor, ClpC (19), while ClpX independently of ClpP functions as a molecular chaperone (20). To confirm that ClpP and ClpX controls β-lactam resistance via formation of the ClpXP protease, we investigated whether β-lactam resistance is increased in cells that retain ClpX chaperone and ClpCP activity but cannot form the ClpXP protease due to a single amino acid substitution in the ClpP recognition IGF motif of ClpX (21). Indeed, introduction of an I_265_E substitution in the IGF tripeptide of ClpX increased the MICs of JE2 against all tested β-lactams confirming that inactivation of the ClpXP protease enhances the β-lactam resistance level of the clinically important CA-MRSA clone USA300 (Table 1). Specifically, expression of the ClpX_I265E_ variant increased the MICs of oxacillin, cefotaxime, and meropenem ∼8-fold, while causing a nonsignificant 2-fold increase in the MICs of imipenem and cefoxitin. We conclude that ClpXP contributes to cellular processes that determine the β-lactam resistance level of JE2.

**TABLE 1:** β-Lactam susceptibilities of wild-type and mutant strains estimated by MICs (mg/liter)

Antibiotic	MIC (μg ml^−1^)											
	JE2						SA564			8325-4		
	WT	*sle1*	*mecA*	*clpX_I265E_*	*clpX_I265E_*, *sle1*	*clpX_I265E_*, *mecA*	WT	*sle1*	*clpX_I265E_*	WT	*sle1*	*clpX_I265E_*
Oxacillin	32–64	0.5	0.75	>256	0.5	1	0.5	0.5	0.75	0.38	0.25	0.5
Imipenem	0.12	0.12	0.12	0.25	0.25	0.25	0.5	0.12	0.12	0.12	0.12	0.12
Cefotaxime	8	2–4	2	64	2	4	4	2	4	1	1	1
Cefoxitin	16	4	4	32	4	4	4	4	4	2	2	4
Cefepime	4	2	2	>32	4-8	4	4	4	4	2	1	2
Ertapenem	0.5	0.12	0.25	>2	0.12	0.25	0.5	0.12	0.25	0.12	0.12	0.25
Meropenem	0.25	0.12	0.12	2	0.12	0.12	0.12	0.06	0.25	0.12	0.06	0.12

### Sle1 is conferring increased β-lactam resistance in JE2 lacking ClpXP activity.

In S. aureus, the cell wall amidase Sle1 is a substrate of the ClpXP protease, and consequently the cellular levels of Sle1 are elevated in cells lacking ClpXP activity (21). To investigate whether the high Sle1 levels play a role in the hyper-resistant phenotype of cells expressing the ClpX_I265E_ variant, we next inactivated *sle1* in JE2 WT and *clpX_I265E_* cells and assessed the impact on β-lactam MICs. Interestingly, inactivation of *sle1* not only abrogated the increased resistance of cells lacking ClpXP protease activity but also decreased MICs below the wild-type level (Table 1). Similarly, inactivation of *sle1* in the JE2 wild type decreased the MICs of all β-lactams except imipenem and rendered JE2 hypersusceptible to oxacillin, with the oxacillin MIC decreasing from 32 μg ml^−1^ in wild-type cells to 0.5 μg ml^−1^ in *sle1* cells. In fact, inactivation of *sle1* rendered cells as susceptible to oxacillin, as did inactivation of *mecA* (Table 1). JE2mecA cells expressing the ClpX_I265E_ variant were as susceptible to β-lactams, as were JE2mecA expressing wild-type ClpX, demonstrating that high Sle1 levels only confer resistance to cells expressing PBP2a. This result is consistent with previous results showing that neither deletion of *clpP* nor deletion of *clpX* alter the MICs of β-lactams in methicillin-sensitive S. aureus (MSSA) strains (5); in agreement with this finding, introduction of the *sle1* and *clpX_I265E_* mutations into the two MSSA strains, SA564 (clinical isolate) and 8325-4 (laboratory strain), had only a slight impact on the MICs (Table 1).

Standard MIC assays prescribe the use of stationary cells, so we sought to determine whether Sle1 levels also impact the ability of exponentially growing JE2 cells to form colonies in the presence of different concentrations of β-lactams. Consistent with the MIC tests, JE2clpX_I265E_ cells were capable of forming colonies in the presence of antibiotic concentrations that inhibited growth of JE2 wild-type cells for all tested β-lactams (see Fig. S1 in the supplemental material). Furthermore, inactivation of *sle1* rendered both wild-type and *clpX_I265E_* cells hypersusceptible to all tested β-lactams (Fig. S1).

We conclude that Sle1 is essential for β-lactam resistance in the JE2 MRSA strain and that ClpXP contributes negatively to β-lactam tolerance via degradation of Sle1.

### Population analysis profiles reveal that *sle1* cells become homogenously hypersusceptible to oxacillin.

**FIG 1:**
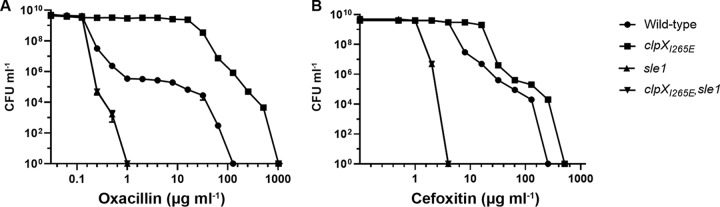
PAPs show that inactivation of *sle1* renders the JE2 wild-type and JE2clpX_I265E_ homogenously susceptible to oxacillin and cefoxitin. The CFU ml^−1^ for JE2 wild-type, JE2clpX_I265E_, JE2sle1, and JE2clpX_I265E_,sle1 strains were determined after plating on increasing concentrations of oxacillin (A) and cefoxitin (B), as indicated. Representative data from three individual experiments are shown.

Similar to other CA-MRSA strains, JE2 displays heterogeneity with respect to β-lactam susceptibility, meaning that the majority of cells exhibit a low level of antibiotic resistance, whereas a minority of cells are highly resistant (1, 5). In order to determine whether inactivation of Sle1 or ClpXP alters the heteroresistant phenotype of the JE2 strain, a population analysis profile (PAP) was determined. In the PAP analysis, we chose to focus on oxacillin and cefoxitin, since these two compounds represent β-lactams whose MICs were highly and marginally effected, respectively, by expression of the ClpX_I265E_ variant. As expected, the PAP analysis generated a typical heterogeneous profile for the JE2 wild-type strain, with the majority of cells being killed by low concentrations of either oxacillin or cefoxitin, while a small subpopulation was capable of growing at much higher concentrations of antibiotics (Fig. 1). Expression of the ClpX_I265E_ variant not only increased the fraction of JE2 cells able to grow in the presence of medium high levels (16 to 32 μg ml^−1^) of antibiotics by 4 logs but also enabled the most resistant subpopulation to grow at even higher concentrations of antibiotics (Fig. 1). On contrast, inactivation of *sle1* transformed both JE2 wild-type and JE2clpX_I265E_ strains into a homogeneously susceptible strain with all cells in the population being inhibited by very low concentrations of antibiotics (Fig. 1).

### Inactivation of Sle1 delays the onset of daughter cell splitting, while high Sle1 levels accelerate the onset of daughter cell separation.

**FIG 2:**
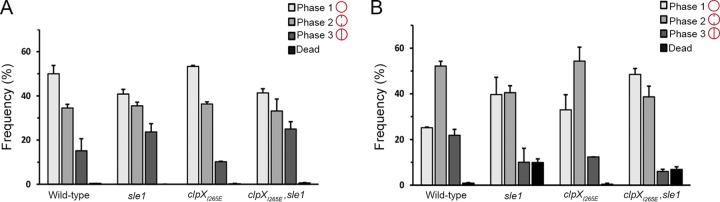
Sle1 and oxacillin impact the S. aureus cell cycle. Exponential cells of JE2 wild-type, JE2clpX_I265E_, JE2sle1, and JE2clpX_I265E_,sle1 strains grown in the absence (A) or presence of oxacillin at 0.05 μg ml^−1^ (B) were stained with the membrane dye Nile Red and imaged with SR-SIM. For each strain, 300 cells were assigned to different phases in the cell cycle based on the stage of septum ingrowth: cells without septal ingrowth were assigned to phase 1, cells displaying an incomplete septum were assigned to phase 2, and cells displaying a closed septum were assigned to phase 3. For each of the strains, the fraction of cells in the three phases of the cell cycle was determined from two biological replicates.

**FIG 3:**
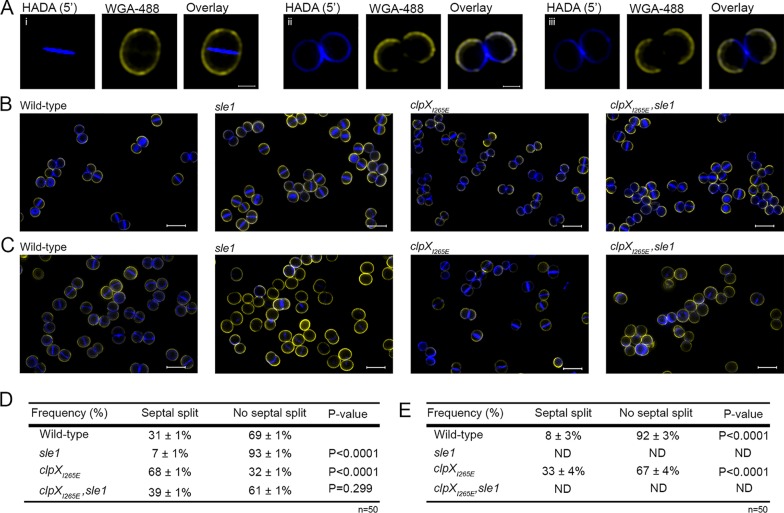
High Sle1 levels accelerate splitting of S. aureus daughter cells, whereas inactivation of Sle1 and oxacillin delay splitting of fully divided daughter cells. SR-SIM images of exponential cells of the JE2 wild-type, JE2sle1, JE2clpX_I265E_, and JE2clpX_I265E_,sle1 strains grown in the absence (A and B) or presence of oxacillin (C) (0.05 μg ml^−1^ for 20 min). Prior to imaging, cells were stained for 5 min with WGA-488 (green) and HADA (blue). (A) Sample images of cells that have completed septum synthesis during the labeling period (closed septal HADA signal) and that have either not separated after labeling (i) or have initiated daughter cell separation after labeling (ii and iii). (D and E) Septal splitting was quantified in 50 cells displaying a closed HADA-stained septum in cells not exposed to oxacillin (D) and in cells exposed to oxacillin (E). The experiment was performed in two biological replicates, and a statistical analysis was performed using a chi-square test comparing either the splitting frequency between mutant and wild-type cells (D) or between cells not exposed or exposed to oxacillin (E).

Based on the finding that deletion of *sle1* induced the formation of cell clusters, it was proposed that Sle1 is involved in separation of S. aureus daughter cells (14). The interesting finding that Sle1 activity impacts resistance to β-lactam antibiotics prompted us to assess the role of Sle1 in S. aureus cell division in more detail using super-resolution structured illumination microscopy (SR-SIM). Prior to SR-SIM, the cells were stained with the membrane stain, Nile Red, and the cell wall stain fluorescent wheat germ agglutinin (WGA-488) that is too big to penetrate into cells and therefore only labels cell wall exposed to the exterior milieu during the staining period (22, 23). To visualize regions of new peptidoglycan insertion, the cells were additionally stained with the blue fluorescent d-amino acid, hydroxycoumarin-amino-d-alanine (HADA). To investigate the impact of Sle1 on the S. aureus cell cycle, we first assigned wild-type and mutant cells to different phases based on the state of septum ingrowth (22): newly separated daughter cells that have not initiated septum formation were assigned to phase 1, and cells in the process of synthesizing division septa were assigned to phase 2, while cells displaying a closed septum were assigned to phase 3. As depicted in Fig. 2A, inactivation of *sle1* significantly increased the fraction of phase 3 cells (*P* = 0.04), while, conversely, the fraction of phase 3 cells was significantly reduced in *clpX_I265E_* cells (*P* = 0.004), although only if the cells express Sle1. Since the percentage of cells observed in each growth phase should be proportional to the fraction of the cell cycle spent in that stage, this finding indicates that separation of fully divided daughter cells is delayed in the absence of Sle1, whereas *clpX_I265E_* cells spend less time in phase 3. In support of this, splitting of the HADA-stained septal wall was only observed in 7% of the *sle1* cells compared to 31% of the wild-type cells and 69% of the cells expressing the ClpX_I265E_ variant (Fig. 3A, B, and D). In conclusion, daughter cell separation is delayed in the absence of Sle1, whereas high levels of Sle1 seem to accelerate the onset of S. aureus daughter cell splitting.

### Sle1 controls cell size.

**FIG 4:**
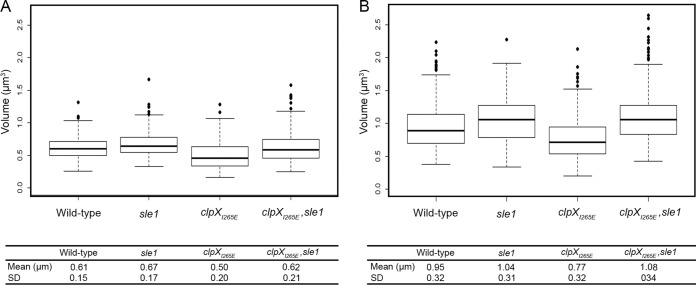
High Sle1 activity reduces the cell size, while oxacillin increases cell size. The cell volume was estimated for JE2 wild-type, JE2clpX_I265E_, JE2sle1, and JE2clpX_I265E_,sle1 cells growing exponentially at 37°C in the absence of oxacillin (A) or after exposure to 0.05 μg ml^−1^ oxacillin for 20 min (B). Cells were stained with the membrane dye Nile Red before imaging by SR-SIM. For each strain, the cell volume was determined in 300 cells representing 100 cells from each of the three growth phases by fitting an ellipse to the border limits of the membrane. The graphs represent data from two biological replicates.

Characterization of the S. aureus cell cycle revealed that S. aureus cells are capable of elongating and that elongation mainly occurs in phases 1 and 3 (22, 23). After establishing that the level of Sle1 impacts the time cells spend in phase 3, we determined whether Sle1 activity impacts the cell size. Indeed, estimation of the cell size demonstrated that *clpX_I265E_* cells are significantly smaller than wild-type cells (*P* < 0.0001), but *clpX_I265E_*, *sle1* cells are similar in size to wild-type cells (Fig. 4A). Inactivation of *sle1* in wild-type cells resulted in cells being slightly, but significantly, larger than wild-type cells (*P* < 0.0001) (Fig. 4A). We conclude that high levels of the Sle1 cell wall amidase leads to a decrease in cell size, while inactivation of Sle1 increases the cell size.

### Scanning electron microscopy indicates that Sle1 contributes to the formation of perforations in the peripheral septal ring prior to popping.

**FIG 5:**
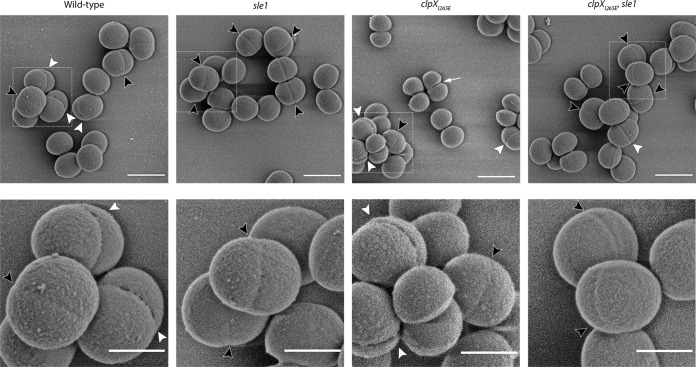
Perforations in the peripheral septal wall correlate with Sle1 levels. SEM images of JE2 wild-type, JE2clpX_I265E_, JE2sle1, and JE2clpX_I265E_,sle1 strains grown in TSB to mid-exponential phase at 37°C. Black arrowheads indicate cells displaying invaginations without cracks at midcell; white arrowheads point to cells displaying cracking along the septal ring at midcell. The white arrow points to a JE2clpX_I265E_ cell that has initiated daughter cell splitting. Boxes in the upper panel are enlarged in the lower panel. Scale bars, 1 μm (upper panel) and 0.5 μm (lower panel).

At the time of cell separation, S. aureus daughter cells are connected only at the edge of the septum by a peripheral ring (23, 24). Resolution of this peripheral wall by mechanical crack propagation results in ultrafast splitting of daughter cells in a process designated “popping” (23). Scanning electron microscopy (SEM) has revealed that popping is preceded by the presence of perforation holes around the bacterial circumference coincident with the outer edge of the division septum, and it was speculated that autolysins are involved in formation of these holes (23). To examine whether Sle1 is the autolysin responsible for creating perforation holes along the septal ring, SEM was used to image the cell surfaces of wild-type and *clpX_I265E_* cells, as well as the surfaces of the corresponding *sle1*-negative strains (Fig. 5). In agreement with published data, small holes are visible at midcell in a small fraction of wild-type cells (Fig. 5). Typically, perforation holes were apparent on ellipsoid cells displaying a slight invagination at midcell, supporting that these cells are in the process of dividing. In support of Sle1 being involved in generating these perforation holes, inactivation of *sle1* in both wild-type and *clpX_I265E_* cells caused the cell wall midcell to appear smoother and less perforated (Fig. 5). On the other hand, the fraction of cells displaying cracks at midcell appeared substantially increased in cells expressing the ClpX_I265E_ variant, and most *clpX_I265E_* cells captured in SEM images are in different stages of cell separation (Fig. 5). These phenotypes of *clpX_I265E_* cells disappeared upon inactivation of Sle1. We conclude that the fraction of cells displaying perforations at midcell correlates to the level of Sle1, indicating that the Sle1 cell wall amidase contributes to degradation of the cell wall in the peripheral ring of the division septum prior to popping.

### Oxacillin treatment impedes separation of daughter cells and interferes with septum formation in cells devoid of Sle1.

**FIG 6:**
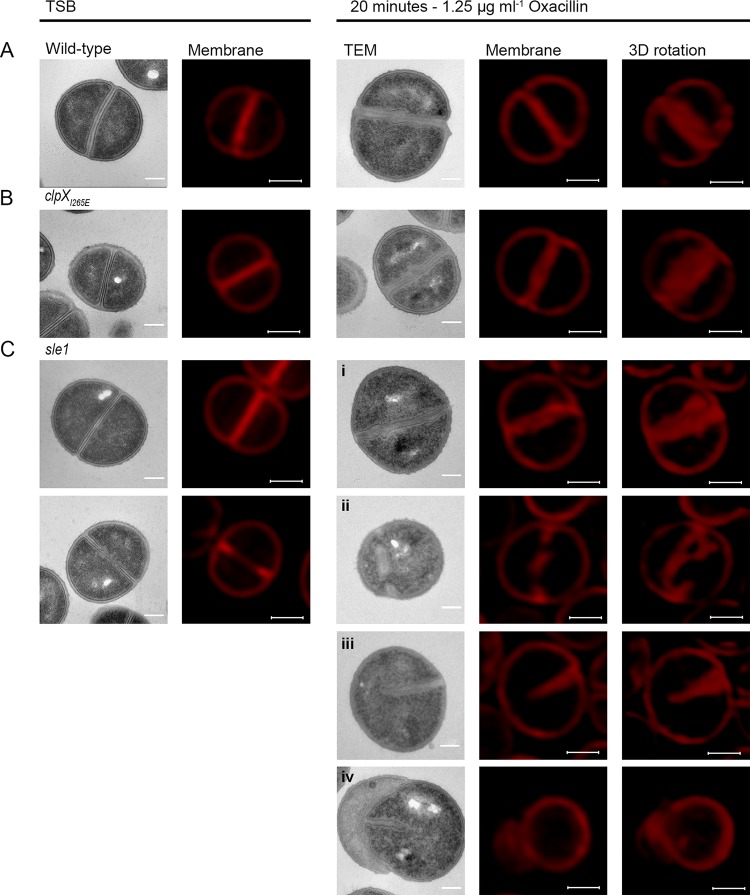
TEM and SR-SIM reveal severe septal abnormalities in *sle1* mutant cells exposed to oxacillin. TEM and SR-SIM images of JE2 wild-type (A), JE2clpX_I265E_ (B), or JE2sle1 (C) strains grown in the absence of oxacillin (left panels) or after exposure to 1.25 μg ml^−1^ oxacillin for 20 min (right panels). The images show the characteristic morphological changes induced by oxacillin as determined from at least two biological replicates. In JE2sle1 cells, oxacillin induces severe septal abnormalities and cell lysis, including thickened septum devoid of the electron-dense midzone (i), a septal fragment that in TEM images appears not to be attached to the peripheral wall (ii), septum protruding asymmetrically inward (iii), and a lysed cell (iv). Scale bars: TEM, 0.2 μm; SR-SIM, 0.5 μm.

We now sought to determine why β-lactam resistance correlates to the expression of Sle1 in the JE2 background. Based on the finding that Sle1 is required for fast separation of S. aureus daughter cells, we first hypothesized that β-lactams impose a cell separation defect that, while being rescued by high Sle1 activity, is lethal to cells lacking Sle1 activity. To test this hypothesis JE2 wild-type, *sle1*, and *clpX_I265E_* cells were exposed to oxacillin at 1.25 μg ml^−1^ for 20 min before the cells were stained with Nile Red, WGA, and HADA and imaged by SR-SIM. Consistent with the idea that oxacillin impedes splitting of daughter cells, the fraction of wild-type cells displaying splitting of the HADA-stained septum was significantly reduced from 31% to <10% (*P* < 0.0001) upon exposure to oxacillin (Fig. 3C and E). Likewise, the fraction of wild-type cells divided by a closed septum (phase 3 cells) and the size of wild-type cells increased significantly after oxacillin exposure (Fig. 2 and 4, respectively). Moreover, high Sle1 levels seem to antagonize the cell separation defect conferred by oxacillin, since significantly more *clpX_I265E_* cells than wild-type cells were capable of splitting in the presence of oxacillin (Fig. 2 and 3). Taken together, these data support that oxacillin confers a cell separation defect that is rescued by high Sle1 activity. On the other hand, oxacillin was expected to exacerbate the cell separation defects of cells devoid of Sle1, but instead oxacillin diminished the fraction of *sle1* cells in phase 3 (Fig. 2), and very few oxacillin-treated *sle1* cells displayed a closed HADA-stained septum (Fig. 3). In fact, the HADA signal was weak or even absent in the majority of *sle1* cells exposed to oxacillin (Fig. 3; see Fig. S2 in the supplemental material). Strikingly, the ability of cells to incorporate HADA in the presence of oxacillin correlated with Sle1 levels, since oxacillin also reduced the HADA signal in wild-type cells, while the intensity of the HADA signal in JE2clpX_I265E_ was similar with or without oxacillin exposure, and did not change, even if cells were treated with higher oxacillin concentrations (Fig. 3; see also Fig. S2). Therefore, oxacillin seems also to impose a septum synthesis defect that is exacerbated in the absence of Sle1. In support of this, the SR-SIM images revealed abnormal septal ingrowths in 75% of *sle1* cells exposed to oxacillin (Fig. 6; see also Fig. S2). To study the morphological changes induced by oxacillin in cells lacking Sle1 in more detail, cells exposed to oxacillin for 20 min were also imaged by transmission electron microscopy (TEM). Both SR-SIM and TEM confirmed severe abnormalities in septal ingrowths in oxacillin-treated *sle1* cells, with septa being devoid of the electron-dense septal midzone previously designated “the splitting line” (25) (Fig. 6Ci to iv; see also Fig. S2 and S3), septa protruding asymmetrically inward (Fig. 6Ciii and iv) and displaying a characteristic “curvy” morphology (Fig. 6Cii to iv). Both TEM and SR-SIM images also revealed that exposure to oxacillin resulted in lysis of a small fraction of *sle1* cells and that lysed *sle1* cells were typically observed in daughter cell pairs, where the lysed cell is attached to a living cell (Fig. 6Civ). Similar, but less severe changes in the septum morphology were observed in wild-type cells exposed to the same concentration of oxacillin (Fig. 6A; see also Fig. S2 and S3). In summary, we found that while high levels of Sle1 seem to enhance tolerance to oxacillin by antagonizing an oxacillin induced cell separation defect, the increased oxacillin sensitivity of *sle1* cells seems to be linked to a synthetic lethal effect on septum synthesis.

### The level of Sle1 is reduced in cells exposed to oxacillin.

**FIG 7:**
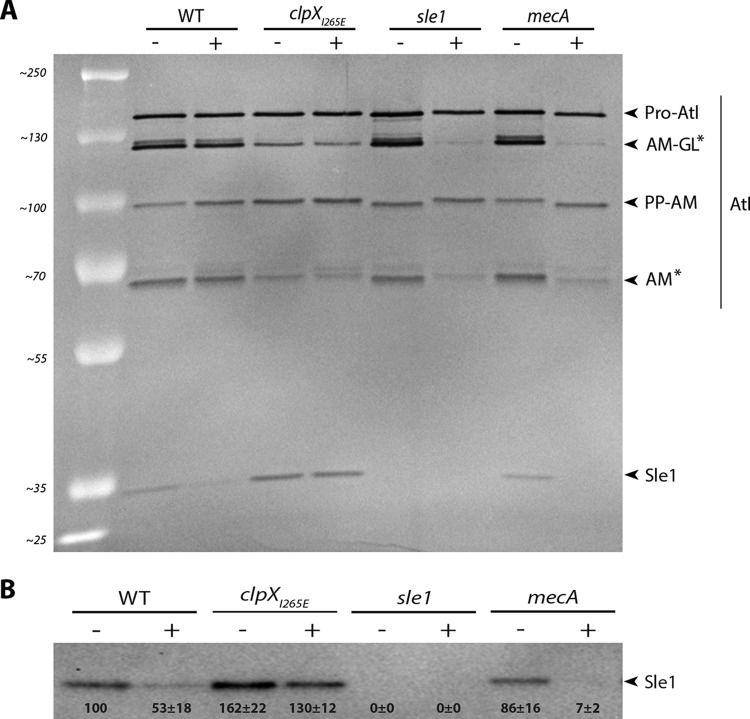
Oxacillin reduces the level of cell wall-associated Sle1, and Sle1 cannot be detected in JE2mecA cells exposed to oxacillin. (A) Zymogram showing cell wall-associated proteins extracted from the indicated strains grown in the absence (–) or presence (+) of 8 μg ml^−1^ oxacillin and then separated on an SDS gel containing heat-killed S. aureus JE2 cells. The inverted image of one representative gel of two biological independent experiments is shown. The positions of the molecular mass standards are indicated on the left (in kilodaltons). Asterisks indicate Atl bands that diminish in oxacillin-exposed JE2 cells lacking Sle1 or PBP2A. (B) Sle1 levels in cell wall extracts were determined by Western blotting in three biological replicates. Densitometry analysis was performed using Fiji. The obtained values were normalized to values obtained for the wild type and are displayed below the corresponding bands.

The two major cell wall hydrolases involved in S. aureus daughter cell splitting are Sle1 and Atl (14, 26, 27). To examine whether oxacillin interferes with splitting of JE2 daughter cells by reducing expression of these enzymes, the murein hydrolase activity was determined in cell wall extracts derived from cells grown in the absence or presence of oxacillin using standard zymography. The resulting murein hydrolase profiles showed multiple bands that were apparently AtlA related, since they disappear in extracts from an Δ*atl* mutant (Fig. 7A; Fig. S4A). Sle1 is a 32-kDa protein comprised of an N-terminal cell wall binding domain and a C-terminal catalytic domain with *N*-acetylmuramyl-l-alanine amidase activity (14). In the zymographs, the activity of the Sle1 autolysin is clearly visible and, as expected, cell wall extracts derived from JE2clpX_I265E_ cells displayed higher Sle1 activity than wild-type cells in both the absence and the presence of oxacillin (Fig. 7A). Interestingly, only the intensity of the Sle1 band was diminished in cell wall extracts derived from JE2 cells exposed to oxacillin (Fig. 7A). These findings indicate that Sle1 expression is downregulated or, alternatively, that export of Sle1 to the cell wall is reduced in JE2 exposed to oxacillin. To distinguish between these two possibilities, we also determined the Sle1 level in cell wall extracts and in whole cells by Western blotting (Fig. 7B; Fig. S4B). In agreement with previous findings, both the nonexported and the exported forms of Sle1 accumulate in JE2 cells expressing the ClpX_I265E_ variant (21). In wild-type cells, however, the nonexported Sle1 was not detected (Fig. S4B), and a similar 2-fold reduction in the Sle1 level was observed in cell wall extracts and in extracts from whole cells (Fig. 7B; see also Fig. S4B). Hence, oxacillin seems to reduce the amount of cell wall-associated Sle1 at the level of synthesis rather than at the level of export.

### Sle1 levels are reduced 10-fold in JE2mecA cells exposed to oxacillin.

The finding that exposure to oxacillin reduced Sle1 levels in JE2 cells shows that the expression of Sle1 is positively coupled to the transpeptidase activity of PBPs. Oxacillin blocks the transpeptidase domain of native PBPs, and we therefore predicted that the expression of Sle1 depends on the transpeptidase activity of PBP2a in oxacillin-treated JE2 cells. To test this hypothesis, we determined the levels of Sle1 in JE2mecA cells grown in the absence or presence of oxacillin. Interestingly, this analysis revealed that, whereas the intensities of Sle1 bands were similar in extracts from wild-type and *mecA* cells grown in the absence of oxacillin, Sle1 was barely detectable in *mecA* cells exposed to oxacillin (Fig. 7). Taken together, these results lend support to the idea that Sle1 expression is coupled to activity of the transpeptidase domain of PBPs and that Sle1 expression becomes dependent on PBP2a activity in JE2 cells exposed to oxacillin.

Finally, we noted that whereas oxacillin did not impact the intensities of the different Atl bands in JE2 wild-type cells, the intensities of two Atl bands were diminished in oxacillin-exposed JE2 cells not expressing Sle1 or PBP2A (Fig. 7A, asterisks). The bifunctional Atl murein hydrolase is produced as a precursor protein (Pro-Atl) that undergoes proteolytic cleavage to yield two catalytically active proteins, an amidase (AM) and a glucosaminidase (GL) (27). The 113-kDa band represents an intermediary cleavage product, whereas the 62-kDa band reflects the activity of the fully cleaved amidase (28). Therefore, PBP2a and Sle1 may also have a role in Atl processing in oxacillin-exposed JE2 cells.

## DISCUSSION

β-Lactam antibiotics are the most frequently prescribed antibiotics worldwide; however, the mechanism by which the binding of β-lactams to their PBP targets causes death and lysis of bacteria is not completely understood (1, 29, 30). In at least some bacteria, the killing mechanism of β-lactams involves unsynchronized activation of peptidoglycan hydrolases (15–18). Contradictive to this model, we show here that the activity of the Sle1 cell wall amidase is crucial for PBP2a-mediated resistance to β-lactams in the JE2 CA-MRSA model strain and that elevated levels of Sle1 confers increased resistance to β-lactams, but only if JE2 expresses PBP2a. The key finding that resistance to β-lactams correlates positively to expression of Sle1 indicates that, in S. aureus, the detrimental effects of β-lactam antibiotics are linked to the inhibition, rather than to the activation, of peptidoglycan hydrolase activity.

Sle1 was proposed to function in S. aureus cell division (14), and with the recent advances in SR-SIM we now examined the role of Sle1 in this fundamental process in more detail using cells either lacking or overproducing Sle1. To divide, S. aureus builds a septal cross wall generating two hemispherical daughter cells that, at the time of cell separation, are connected only at the peripheral ring forming the outer edge of the septum (23, 24). Resolution of this peripheral wall involves mechanical crack propagation, but the contribution of cell wall hydrolases to the ultrafast popping of S. aureus daughter cells remains poorly described (23). We demonstrate that high levels of Sle1 accelerate the onset of daughter cell separation starting from the peripheral wall, indicating that Sle1 contributes to the timely degradation of the outer edge of the septal wall. Sle1, however, is not required for resolution of the outer septal wall, since the separation of daughter cells is delayed but not inhibited in cells lacking Sle1. Previously, cryo-electron microscopy revealed that, at the beginning of septation, the peripheral ring is thicker that other parts of the outer wall, and it was proposed that this extra cell wall material serve to protect the peripheral wall from degradation by the cell wall hydrolases functioning in presplitting of the septal cross-walls (24). Interestingly, the TEM and SR-SIM pictures presented here suggest that Sle1 degrades the peripheral wall from the exterior and not from the interior. Taken together, our data support that high Sle1 levels promote daughter cell splitting, indicating that the detrimental effect of β-lactam antibiotics is linked to impaired daughter cell separation.

We, similarly to others, observed that oxacillin delays cell separation (18, 31, 32) and, based on the characteristic “hour-glass” morphology observed for wild-type cells exposed to oxacillin (indicating that splitting from the cell periphery has taken place [Fig. S3]), we speculate that oxacillin primarily interferes with splitting of the interior septal cross-walls. Interestingly, the electron-dense line that vanishes in oxacillin-exposed cells was previously described as tubular packets enclosing autolytic enzymes that upon completion of the cross wall are released to facilitate cell separation (18, 25).

**FIG 8:**
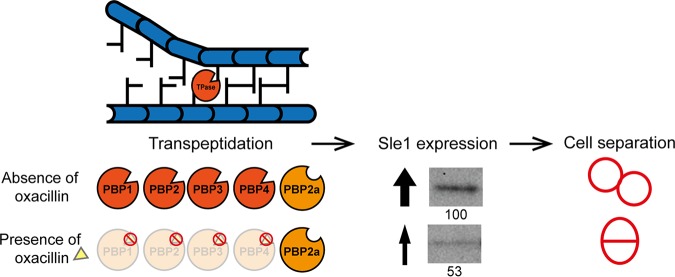
Model showing that the expression of Sle1 is coupled to the TP activity of PBPs. The model predicts that the TP activity of PBPs plays a role in signaling that peptidoglycan synthesis is complete and that it is time to activate the expression of Sle1. In the absence of β-lactam antibiotics, the TP activity of both native PBPs and the *mecA*-encoded PBP2a contribute to the cross-linking of peptidoglycan and to the activation of Sle1 expression. In cells exposed to β-lactam antibiotics, however, the TP activity of native PBPs is inhibited by the irreversible binding of oxacillin, and the expression of Sle1 becomes entirely dependent on the TP activity of PBP2a. PBP2a is less efficient in promoting expression of Sle1 than are the native PBPs, and therefore the Sle1 levels are reduced in JE2 cells exposed to oxacillin. See the text for details.

Next, we showed that oxacillin reduces the expression of Sle1 in JE2 cells, suggesting that oxacillin impairs daughter cell splitting by downregulating Sle1 expression. Interestingly, the Tomasz lab has convincingly shown that transcription of genes encoding cell wall hydrolases is tightly linked to the activities of the PBPs and that the inhibition of PBP activity, either by genetic depletion or by treatment with β-lactam antibiotics, reduces transcription of a number of cell wall hydrolase genes, including *sle1* (33–35). In these studies, the β-lactam-induced transcriptional repression of cell wall hydrolase genes was proposed to be a defense mechanism protecting cells with perturbed cell wall synthesis from the destructive forces of cell wall hydrolases. Based on our paradoxical finding that high levels of Sle1 increase resistance of S. aureus to β-lactam antibiotics, we instead hypothesize that the shutdown of transcription of *sle1* and other cell wall hydrolase genes in S. aureus cells exposed to β-lactam antibiotics is part of the mechanism that eventually end up killing the cells. Instead, we speculate that PBP-mediated transpeptidation, the last step in peptidoglycan synthesis, is involved in signaling that peptidoglycan synthesis is complete and that it is time to activate expression of *sle1* and other cell wall hydrolases (see the model depicted in Fig. 8). According to this model, the TP activity of native PBPs and PBP2a can both fulfill this role. In cells exposed to β-lactam antibiotics, however, the transpeptidase activity of native PBPs is inhibited by the irreversible binding of oxacillin to the active site, and therefore the expression of Sle1 becomes entirely dependent on the TP activity of the PBP2a (Fig. 8). Consistent with this model, we show here that whereas PBP2a is required for the expression of Sle1 in JE2 cells exposed to oxacillin, the deletion of *mecA* does not impact Sle1 levels in JE2 cells grown in the absence of oxacillin. The observation that Sle1 levels are reduced 2-fold upon exposure to oxacillin indicates that the nonnative PBP2a is less efficient in promoting expression of Sle1 than are the native PBPs (Fig. 8). At present we do not know whether the expression of Sle1 is specifically linked to the activity of one or more of the native PBPs; however, previous data indicate that transcription of the *sle1* gene is coupled to the activity of both PBP1 and PBP2 (34, 35). In S. aureus, at least 13 genes encode known or putative peptidoglycan hydrolases (36). Strikingly, transcription of many of these genes responds to PBP activity in a strain-dependent manner (33–35) and thus may also respond to PBP2a activity in a strain-dependent manner, thereby contributing to the strain-dependent resistance level conferred by *mecA*.

Finally, we found that inactivation of *sle1* is synthetically lethal with sub-MICs of oxacillin in JE2. At present, we cannot explain this finding, but the severe septum defects conferred by oxacillin in cells devoid of Sle1 activity indicate that Sle1 and the transpeptidase activity of PBPs function synergistically to coordinate septum formation with daughter cell separation. Consistent with this finding, Matias and Beveridge (24) proposed that cell wall autolysins are required for synchronized growth of the septum. Intriguingly, a recent report suggests that another S. aureus cell wall amidase, LytH, is involved in controlling the spatial distribution of peptidoglycan synthases to ensure that cell expansion is coordinated with cell division (37). Hence, the activities of cell wall hydrolases and PBPs may be tightly linked to balance peptidoglycan synthesis with autolytic degradation, and the binding of β-lactam antibiotics PBPs may perturb this delicate balance.

## MATERIALS AND METHODS

### Bacterial strains and growth conditions.

The bacterial strains used in this study are listed in Table 2. S. aureus JE2, SA564, and 8325-4 were used as wild-type strains. Strains were cultured in 20 ml of tryptic soy broth (TSB; Oxoid) with shaking (170 rpm) at 37°C or on tryptic soy agar (TSA; Oxoid) at 37°C. In all experiments, bacterial strains were freshly streaked from the frozen stocks on TSA and incubated overnight at 37°C. From these plates, TSB cultures were inoculated to an optical density at 600 nm (OD_600_) of ≤0.05, and the OD_600_ values were determined.

**TABLE 2:** Bacterial strains used in the present study

Strain	Description	Source or reference
JE2	CA-MRSA strain USA300 LAC cured of plasmids	38
JE2sle1	*sle1*::ΦΝΣ transduced from NE1688 (38) into JE2 wild-type	This study
JE2clpX_I265E_	*clpX_I265E_* variant from 8325-4 *clpX_I265E_* transduced into JE2 wild type	This study
JE2clpX_I265E_,sle1	*sle1*::ΦΝΣ transduced from NE1688 (38) into JE2 *clpX_I265E_*; *ermB*	This study
JE2mecA	*mecA*::ΦΝΣ transduced from NE1868 (38) into JE2 wild-type; *ermB*	This study
JE2clpX_I265E_,mecA	*mecA*::ΦΝΣ transduced from NE1868 (38) into JE2 *clpX_I265E_*; *ermB*	This study
SA564	Low-passage clinical isolate	40
SA564sle1	*sle1*::ΦΝΣ transduced from NE1688 (38) into SA564 wild type; *ermB*	This study
SA564clpX_I265E_	SA564 expressing a ClpX_I265E_ variant from the native *clpX* locus	21
8325-4	Widely used wild-type strain cured of all prophages	41
8325-4sle1	*clpX_I265E_* variant from 8325-4 *clpX_I265E_* transduced into 8325-4 wild type; *ermB*	This study
8325-4clpX_I265E_	8325-4 expressing a ClpX_I265E_ variant from the native *clpX* locus	21

### Construction of strains.

Sle1 and PBP2a were inactivated by introducing *sle1*::ΦΝΣ from NE1688 or *mecA*::ΦΝΣ from NE1868 (38), respectively, using phage 85 and selecting for resistance to erythromycin. In order to construct a JE2clpX_I268E_, *sle1* double mutant, JE2 chromosomal *clpX* was first replaced with an untagged version of *clpX_I265E_* (substituting the ATT codon with GAA) by phage 85 mediated transduction using 8325-4clpX_I265E_ (21) as the donor. In order to select for JE2 cells that had incorporated the markerless *clpX_I265E_* variant, we took advantage of the finding that expression of the ClpX_I265E_ variant increases the resistance of JE2 to oxacillin by plating transductants at 50 μg ml^−1^ oxacillin. Introduction of the *clpX_I265E_* variant was subsequently confirmed using a primer pair designed to distinguish wild-type *clpX* from *clpX_I265E_* (the 3′ end is complementary to the mutated GAA codon, sequence underlined in primer: clpX_I265E__F (5′-CGT CTT GGT GAA AAA GTT GAA) and clpX_R (5′-CCG TGG CTA GCA TGT TTA AAT TCA ATG AAG A). To ensure that the high oxacillin concentration had not introduced additional mutations in the genome of the JE2clpX_I265E_ candidate, the genomes of the JE2 wild-type and JE2clpX_I265E_ candidate strains were sequenced by Illumina sequencing on a NextSeq instrument at the Danish National Reference Laboratories for Resistance Surveillance (SSI, Copenhagen, Denmark). All sequence analysis was performed in CLC Genomics Workbench Software (v12.0; https://www.qiagenbioinformatics.com). The sequencing reads from the JE2 wild type and the JE2clpX_I265E_ candidate were mapped to USA300 FPR3757 chromosomal genome sequence (GenBank accession number NC_007793.1). Sequencing reads are uploaded at the European Nucleotide Archive under study accession number PRJEB34544. Genomic variations were identified using the “basic variant detection” tool in CLC. This analysis confirmed the introduction of the GAA codon in the JE2clpX_I265E_ candidate and identified three additional single nucleotide polymorphisms (SNPs) between the JE2 wild-type sequence and the sequence of the JE2clpX_I265E_ candidate: one SNP was found in a noncoding region close to *clpX*, while the two other SNPs were mapped in the *hemA* gene that is located one gene downstream of *clpX*. The *hemA* sequence in the JE2_clpXI265E_ candidate is identical to the *hemA* sequence in strain 8325-4, suggesting that all identified SNPs originate from 8325-4clpX_I265E_ that was used as the donor in the transduction.

### Susceptibility testing.

Susceptibility testing was performed by the Danish National Reference Laboratories for Resistance Surveillance (SSI) using Etest (bioMérieux) to determine the MICs of oxacillin and including S. aureus strain ATCC 43300 as a reference strain. The Sensititre Vizio broth microdilution system (Thermo Fisher Scientific) was used to determine the MICs of all other antibiotics using S. aureus strain ATCC 29213 as a reference strain.

### Population analysis profiles.

Population analysis profiles were determined by plating appropriate dilutions of an overnight S. aureus culture on TSA plates containing increasing concentrations of oxacillin or cefoxitin (Sigma). Plates were incubated at 37°C for 48 h, and the number of colonies was determined and plotted against antibiotic concentration as described previously (39).

### SR-SIM analysis. (i) Imaging and sample preparation.

For SR-SIM analysis, cells were imaged with an Elyra PS.1 microscope (Zeiss) using a Plan-Apochromat 63×/1.4 oil DIC M27 objective and a Pco.edge 5.5 camera. Images were acquired with five grid rotations and reconstructed using ZEN software (black edition, 2012, v8.1.0.484) based on a structured illumination algorithm, using synthetic, channel-specific optical transfer functions and noise filter settings ranging from −6 to −8. Laser specifications can be found in Table 3. Prior to imaging, cultures of S. aureus were grown at 37°C for four generations before dividing the cultures into two: one grown in the absence of oxacillin and the other supplemented with oxacillin at 1.25 μg ml^−1^. Cultures where grown for 20 min before sample preparation. Cells were stained at 37°C for 5 min with the membrane dye Nile Red, the cell wall dye WGA-488, and the fluorescent d-amino acid HADA (Table 3). Samples were washed twice in phosphate-buffered saline (PBS), placed on an agarose pad (1.2% in PBS), and visualized by SR-SIM, as described above. SR-SIM analysis was performed at the Core Facility of Integrated Microscopy (CFIM).

**TABLE 3:** Staining and laser specifications used for SR-SIM

Staining	Concn	Target	Laser (nm)	Laser type, mW	Laser power (%/ms)	Beam splitter	Grating (μm)
Nile Red	5 μg ml^−1^	Membrane	561	HR diode, 100	5/100	BP 570-650 + LP 750	34
HADA	250 μM	New PG	405	HR diode, 50	20/100	BP 420-480 + LP 750	23
WGA-488	1 μg ml^−1^	Old PG	488	HR diode, 100	5/100	BP 495-575 + LP 750	28

### (ii) Analysis of the cell cycle.

To address progression of the cell cycle, 300 cells were scored according to the stage of septum ingrowth: no septum (phase 1), incomplete septum (phase 2), or nonseparated cells with complete septum (phase 3). Scoring was based on the Nile Red staining. Dead cells were scored as collapsed cells with no HADA incorporation. To enumerate the fraction of phase 2 cells displaying symmetric septal ingrowth versus abnormal ingrowths, 100 Nile Red-stained cells displaying septal ingrowths were evaluated in each of two biological replicates. To quantify daughter cell splitting, 50 cells that had completed septum formation during the 5 min of HADA labeling were scored based on whether they displayed no septal splitting as depicted in Fig. 3Ai or septal splitting as depicted in figure Fig. 3Aii and iii. This analysis was performed for two biological replicates.

### (iii) Estimating cell volume (size).

The volume of 300 cells representing 100 cells from each of the three different growth phases was determined (two biological replicates). An equal number of cells from each phase was used in order to avoid bias in average volume due to a shift in the phase distribution. Volume was determined as described previously (22). Briefly, an ellipse was fitted to the border limits of the membrane to acquire measurements of the minor and major axis. The cell shape was assumed to be that of a prolate spheroid, and the volume was estimated by using the equation *V* = 4/3π*ab*^2^, where *a* and *b* correspond to the major and minor axes, respectively. Ellipse fitting and measurements were performed using Fiji (http://fiji.sc).

### Statistical analysis.

All statistical analyses were performed using R statistical software. A Student *t* test was used to assess significant differences in cell volume. A chi-squared test of independence was used to determine whether there was a significant relationship between the proportion of cells assigned to each of the three phases or relevant phenotypes under the tested condition (number of cells in the relevant phase or phenotype/the total number of cells). A *P* value of <0.05 was considered significant.

### Scanning electron microscopy.

Strains were grown in TSB at 37°C, as specified, with an initial starting OD of 0.02. Exponentially growing cells were collected and placed on ice for 5 min prior to centrifugation (13,400 rpm; 1 min). Cell pellets were resuspended in fixation solution (2% glutaraldehyde in 0.05 M sodium phosphate buffer [pH 7.4]) and deposited on the glass disks at 4°C for a minimum of 24 h. The specimens were subsequently washed three times in 0.15 M sodium phosphate buffer (pH 7.4) and postfixed in 1% OsO_4_ in 0.12 M sodium cacodylate buffer (pH 7.4) for 2 h. After a rinse in distilled water, the specimens were dehydrated to 100% ethanol according to standard procedures and critical point dried (Balzers CPD 030) with CO_2_. The specimens were subsequently mounted on stubs using double adhesive carbon tape (Ted Pella) as an adhesive and sputter coated with 6-nm gold (Leica ACE 200). SEM observations were performed using a FEI Quanta 3D scanning electron microscope operated at an accelerating voltage of 2 kV.

### Transmission electron microscopy.

Strains were grown in TSB at 37°C as specified above, but with an initial OD_600_ of 0.02, and at an OD_600_ of 0.2 the culture was divided in two: one without oxacillin and one supplemented with oxacillin at 1.25 μg ml^−1^. The cultures were incubated at 37°C for 20 min. The cells were collected by centrifugation (8,000 × *g*; 5 min) and suspended in fixation solution as described above, followed by incubation overnight at 4°C. The fixed cells were further embedded in agarose, rinsed three times in 0.15 M sodium phosphate buffer (pH 7.2), and subsequently postfixed in 1% OsO_4_ with 0.05 M K_3_Fe(CN)_6_ in 0.12 M sodium phosphate buffer (pH 7.2) for 2 h. The specimens were dehydrated in a graded series of ethanol, transferred to propylene oxide, and embedded in Epon according to standard procedures. Sections, approximately 60 nm thick, were cut with an Ultracut 7 (Leica, Wienna, Austria) and collected on copper grids with Formvar supporting membranes, stained with uranyl acetate and lead citrate. Specimens examined with a Philips CM 100 transmission EM (Philips, Eindhoven, The Netherlands), operated at an accelerating voltage of 80 kV. Digital images were recorded with an Olympus Veleta digital slow scan 2.048 × 2.048 charge-coupled device camera and the ITEM software package.

All SEM and TEM processing and microscopy of fixed cells were performed at the CFIM.

### Western blotting.

S. aureus cultures were inoculated in TSB at 37°C and grown for four generations. Cultures were divided into two: one without oxacillin and one supplemented with oxacillin at 8 μg ml^−1^. The cultures were further incubated at 37°C for 60 min, and the cells were harvested at an OD_600_ of 1 to 2. Cell wall-associated proteins were extracted by resuspending pellet in 4% SDS (normalized to an OD of 1 ml^−1^), followed by incubation for 45 min at room temperature with gentle shaking as described previously (39). For immunoblotting, samples were loaded on NuPAGE 10% Bis-Tris gels (Invitrogen) using morpholinepropanesulfonic acid buffer (Invitrogen). After separation, proteins were blotted onto a polyvinylidene difluoride membrane using an Invitrolon polyvinylidene difluoride filter paper sandwich (0.45-μm pore size; Invitrogen). Membranes were preblocked with human IgG to avoid a protein A signal. The Sle1 protein was detected using rabbit-raised antibodies against staphylococcal Sle1 (14). Bound antibody was detected with the WesternBreeze chemiluminescent anti-rabbit kit. Densitometry analysis for three biological replicates was performed using the ImageJ gel analysis tool, where the background from the gel was removed individually for each band.

### Zymographic analysis.

Bacteriolytic enzyme profiles were obtained using the protocol described in reference 42. Autolytic enzyme extracts were prepared by growing bacterial strains as described for Western blotting. Then, 10-ml portions of culture were withdrawn and washed twice with 1 volume of ice-cold cold 0.9% NaCl. Cell wall-associated proteins were extracted by resuspending pellet in 1 ml of 4% SDS (normalized to an OD of 1 ml^−1^), followed by incubation for 45 min at room temperature with gentle shaking. The cells were precipitated (8,000 rpm; 5 min), and the supernatant was used as a source of enzymes. Proteins were separated by SDS-PAGE using a 10% resolving gel containing 1% heat-killed JE2 wild-type cells. After electrophoresis, the gel was washed with ionized water for 15 min three times and subsequently incubated for 20 to 24 h in renaturing buffer (50 mM Tris-HCl [pH 7.5], 0.1% Triton X-100, 10 mM CaCl2, 10 mM MgCl_2_) at 37°C with gentle agitation. The gel was rinsed in ionized water, stained (0.4% methylene blue, 0.01% KOH, 22% ethanol) for 1 min, and destained with ionized water for 1 h with gentle agitation prior to photography.

## Supplementary Material

Supplemental file 1
