# Supplementary material for: The Sle1 Cell Wall Amidase Is Essential for β-Lactam Resistance in Community-Acquired Methicillin-Resistant Staphylococcus aureus USA300
Source: Antimicrob Agents Chemother. 2019 Dec 20;64(1):e01931-19. doi: 10.1128/AAC.01931-19 (PMC7187620; doi:10.1128/AAC.01931-19)
Supplement: Supplemental file 1 [file AAC.01931-19-s0001.pdf]

Supplemental material

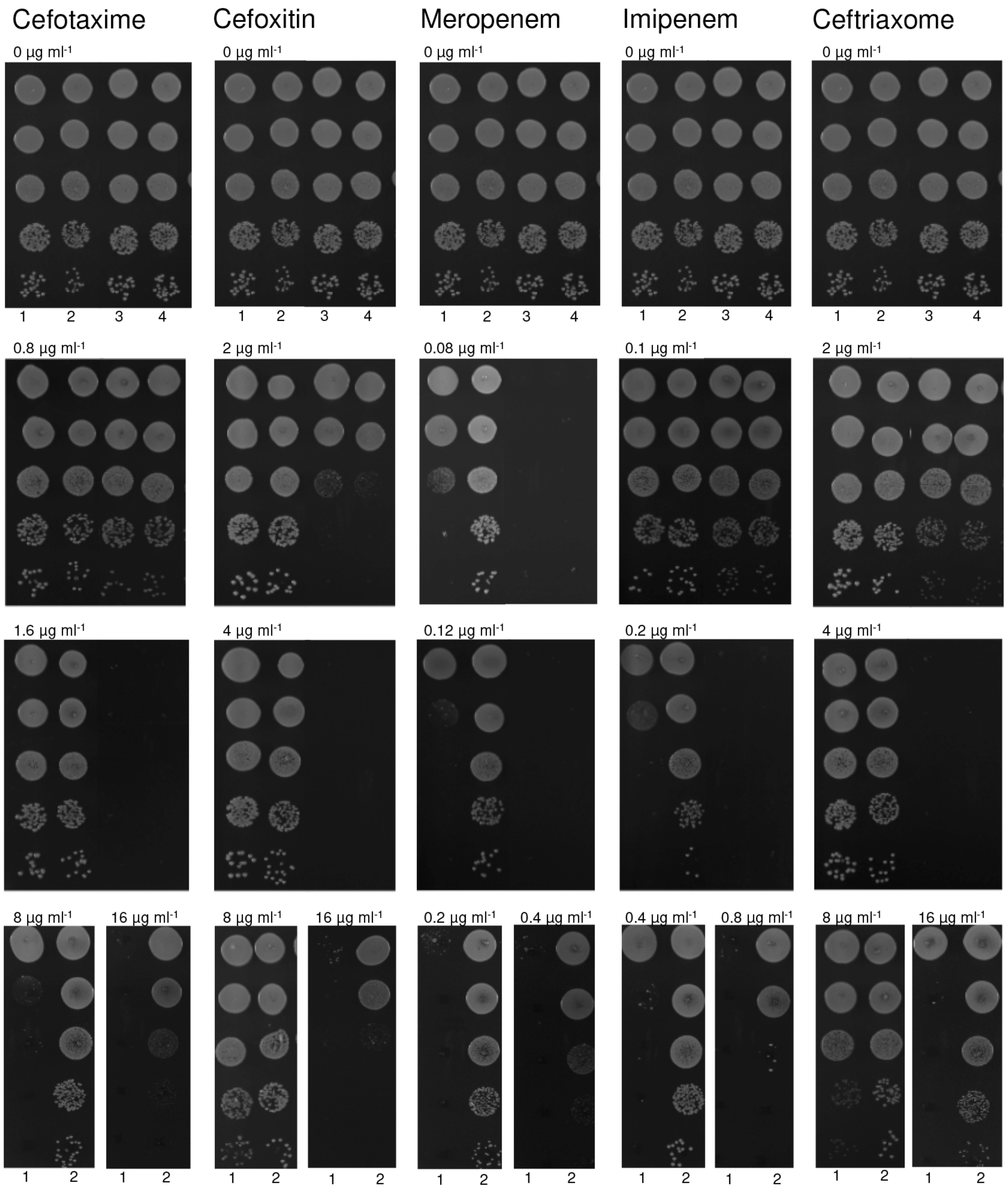

Fig. S1 β-lactam susceptibility in JE2 cells is correlated with Sle1 levels. *S. aureus* JE2 wild-type (1), *sle1* (2), *clpX*$_{I265E}$ (3) and *clpX*$_{I265E}$ *sle1* (4) cells were grown exponentially in TSB at 37°C. At OD$_{600}$ = 0.5, cultures were diluted 10^1, 10^2, 10^3 and 10^4-fold. 10 µl of 10^0 and each dilution were spotted on TSA plates with or without β-lactams as indicated.

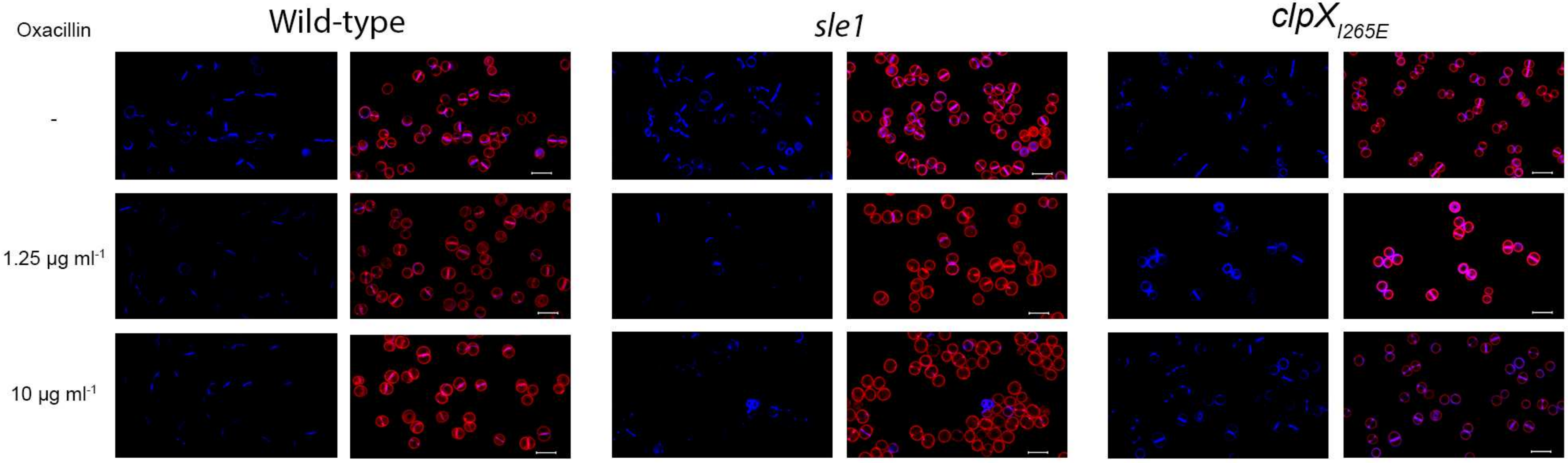

Fig. S2 Oxacillin interferes with peptidoglycan synthesis in cells devoid of Sle1. SR-SIM images of JE2 wild-type, *clpX*$_{I265E}$, or *sle1* cells grown to mid-exponential phase at 37°C in the absence or presence of 1.25 µg ml^{-1} or 10 µg ml^{-1} oxacillin for 20 minutes as indicated.

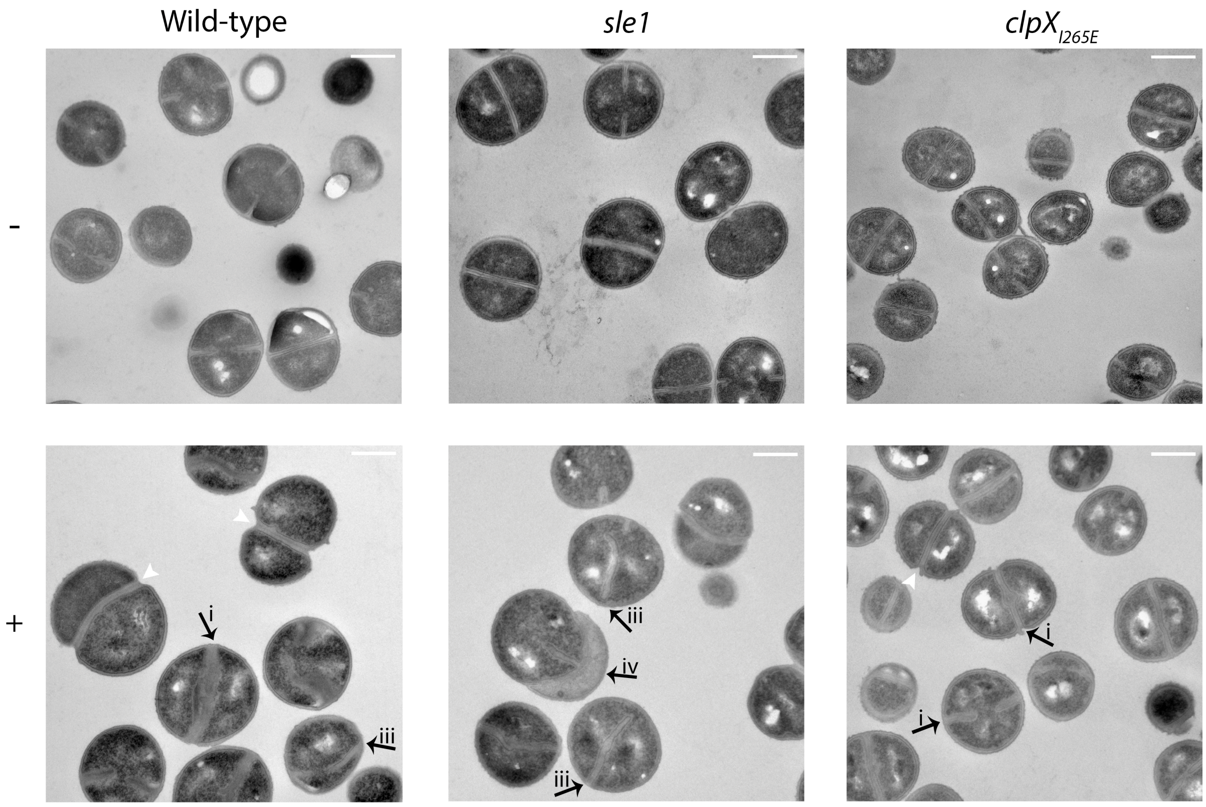

Fig. S3 TEM images showing morphological changes in cells exposed to oxacillin. TEM images of JE2 wild-type, *sle1* or *clpX*$_{I265E}$ cells grown in TSB to mid-exponential phase at 37°C in the absence (-) or presence of 1.25 µg ml^{-1} oxacillin (+) for 20 min. Black arrows point to the characteristic morphological changes described in the legend to Fig. 6: i) thickened septum devoid of the electron-dense mid-zone, iii) septum protruding asymmetrically inwards, and iv) lysed cell. Scale bars, 0.5 µm.

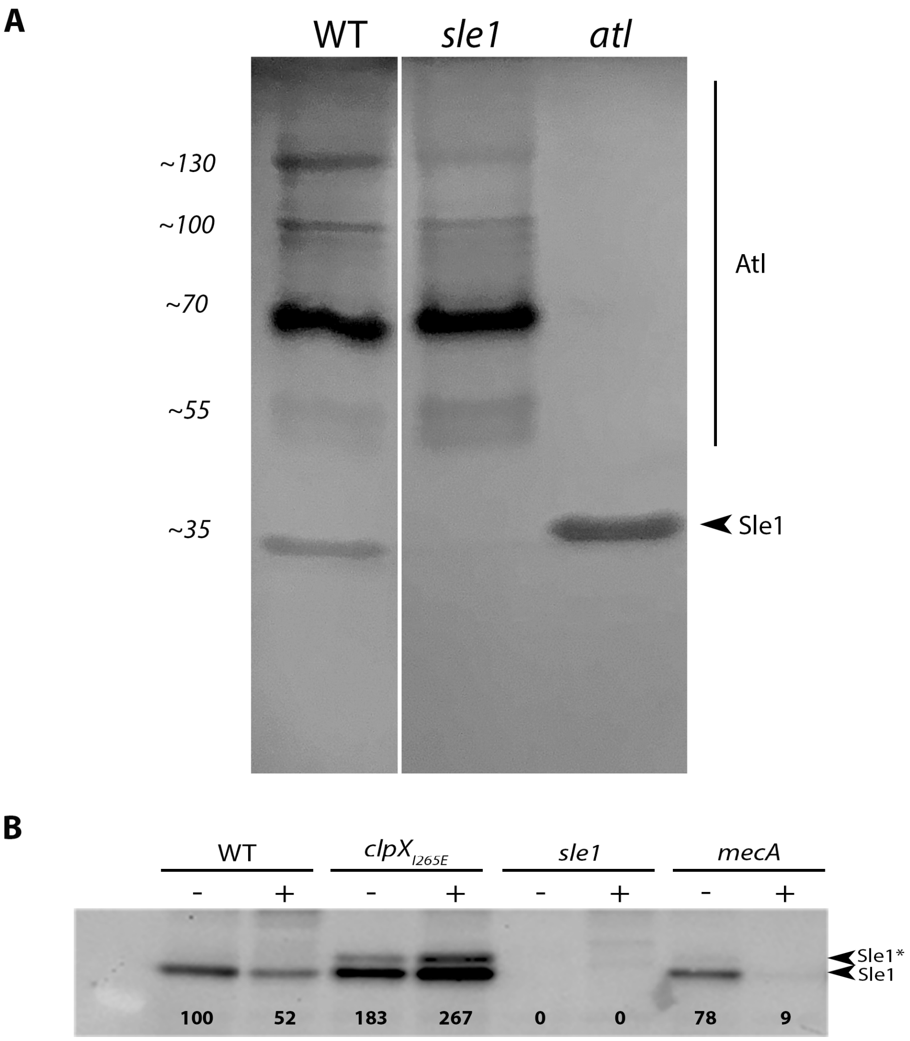

Fig S4. Sle1 does not accumulate intracellularly in cells exposed to oxacillin. (A) Zymography demonstrating that the activity of Atl is visible in multiple bands reflecting that the bifunctional Atl murein hydrolase is produced as a precursor protein (Pro-Atl) that is sequentially cleaved to generate several intermediates (B) Sle1 levels in whole cell extracts from the JE2 wild-type wild type and mutant cells grown at 37°C in the absence (-) or presence (+) of 8 µg/ml oxacillin for 1 hour were determined by Western blotting using a Sle1 specific antibody. The Sle1 levels in the different strains were normalized to wild-type levels (100%) using ImageJ. In extracts derived from JE2clpX$_{I265E}$ cells, the Sle1 antibody specifically recognizes two bands that presumably represent Sle1 with (Sle1*) and Sle1 without a signal peptide.
